# Platelet-Derived Microparticles and Autoimmune Diseases

**DOI:** 10.3390/ijms241210275

**Published:** 2023-06-17

**Authors:** Xiaoshuai Li, Qiushi Wang

**Affiliations:** Department of Blood Transfusion, Shengjing Hospital of China Medical University, Shenyang 110801, China; 2019140168@stu.cmu.edu.cn

**Keywords:** microparticles, platelet-derived microparticles, autoimmune pathogenesis, autoimmune diagnosis, autoimmune treatment

## Abstract

Extracellular microparticles provide a means of cell-to-cell communication and can promote information exchanges between adjacent or distant cells. Platelets are cell fragments that are derived from megakaryocytes. Their main functions are to stop bleeding, regulate inflammation, and maintain the integrity of blood vessels. When platelets are activated, they can perform related tasks by secreting platelet-derived microparticles that contain lipids, proteins, nucleic acids, and even organelles. There are differences in the circulating platelet levels in many autoimmune diseases, including rheumatoid arthritis, systemic lupus erythematosus, antiphospholipid antibody syndrome, and Sjogren’s syndrome. In this paper, the latest findings in the research field of platelet-derived microparticles are reviewed, including the potential pathogenesis of platelet-derived microparticles in various types of immune diseases, their potential as related markers, and for monitoring the progress and prognosis of disease treatment are expounded.

## 1. Introduction

Extracellular microparticles (MPs) provide a means of cell-to-cell communication and can promote information exchanges between adjacent or distant cells [1,2]. Among all the types of MPs in the blood of healthy individuals, platelet-derived microparticles (PMPs) are the most abundant [3,4]. Autoimmune diseases are pathological autoimmune reactions caused by many factors, which are mainly manifested in the destruction and injury of their own tissues and cellular components through the autoimmune system, ultimately leading to tissue damage and organ dysfunction [5,6,7]. Autoimmune diseases include rheumatoid arthritis, systemic lupus erythematosus, Sjogren’s syndrome and various others, which usually involve the blood, joints, muscles, bones and soft tissues around the joints [5,6,7]. Once a patient is diagnosed, the autoimmune disease should be treated in time to avoid further development of the disease and damage to tissues, organs, or systems. Therefore, it is important to monitor and distinguish between various autoimmune diseases. During the occurrence and development of autoimmune diseases, the level of PMP in the body will change, which suggests that PMP can be used as a potential biomarker for the detection of autoimmune diseases and can be used as a biomarker for monitoring the therapeutic response and prognosis of autoimmune diseases in the future. At present, the research field of PMP in certain autoimmune diseases is limited; therefore, this article reviews the research hotspots in the field of PMPs, and discusses their potential as a mechanism in the treatment process and potential significance in diagnosis and treatment of autoimmune diseases.

## 2. Overview of PMPs

In 1967, Peter Wolf discovered a component derived from platelets that could participate in blood coagulation [8]. This tiny component has been proven to be an extracellular vesicle produced when platelets are activated [8,9]. Further research has shown that activated platelets release two types of vesicles: exosomes (30–150 nm) derived from multivesicular bodies of the endocytic tract, and PMPs (100–1000 nm) are shed by the budding of the plasma membrane [10,11]. Although there are apparent differences between platelet-derived exosomes and PMPs in terms of shape, origin, and function (Table 1), understanding of the two has often been confused. With the continuous progress in vesicle detection and separation technology, it is now possible to distinguish and separate platelet-derived exosomes and PMPs. Currently, research hotspots in various fields are focused on exosomes, and in-depth research on PMPs is relatively limited. PMPs represent the most abundant MPs in human circulation [12,13,14]. Many studies have recognized the role of PMPs in hemostasis, thrombosis, cardiovascular disease, autoimmune diseases and cancer [15,16,17,18,19]. However, the role of PMPs in autoimmune diseases has not yet been reviewed in detail.

### 2.1. Formation of PMPs

**Figure 1:**
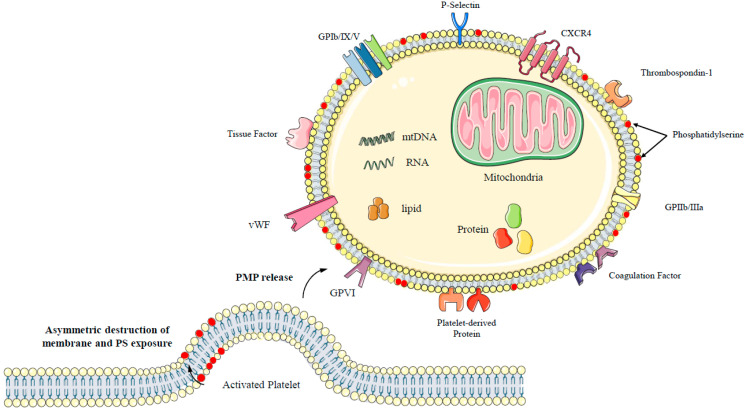
Diverse components are present in the PMPs. Although PMPs are derived from platelets, various molecules and organelles are present either on or inside them. PMP cargo includes nucleic acids, lipid mediators, mitochondria, and proteins (enzymes, transcription factors, receptors, cytokines, etc.). Note that different platelet-activating pathways or experimental parameters may affect PMP content.

Although research on the synthesis and secretion mechanisms of PMPs has made significant progress after nearly 30 years of development, the exact mechanism involved in PMP output has not been clearly defined. It has been proven that the production of PMPs can be triggered via several means: (1) platelet activation via soluble agonists, (2) shear stress, or (3) glycoprotein (GPIIb/IIIa) outside-in signal transduction. After platelet activation, intracellular calcium ions continuously increase [20,21], which stimulates the activation of various phospholipases to maintain the activity of normal platelet plasma membrane skeleton proteins. Some plasma membranes lose support after the skeleton proteins move, forming vacuoles protruding outwards and forming pseudopodia. The phospholipid arrangement in the platelet plasma membrane is asymmetric; the outer layer mainly consists of phosphatidylcholine (PC) and sphingomyelin (SM), whereas the inner layer consists of phosphatidylserine (PS). The arrangement of the plasma membrane structure is regulated and balanced by invertase, aminophospholipid transposase, floppase, and scramblase [22]. Platelet activators, such as thrombin, collagen, and calcium ions, can promote or inhibit the action of these enzymes and mediate the activities of platelet membrane phospholipids and skeleton proteins. Platelet activators are mainly manifested by the exposure of negatively charged phospholipids (such as PS) to the outer leaf of the plasma membrane, when the part of the plasma membrane forming pseudopodia protrudes outward and forms a PMP incorrectly [22] (Figure 1).

### 2.2. Composition of PMPs

The surface markers of PMPs vary in different diseases, and the contents of PMPs are complex and diverse [23,24]. At present, it is generally believed that membrane disorder and PS exposure are the key factors promoting PMP release, however, most of the PMP surfaces in the blood, lymph, and synovial fluid of patients with rheumatoid arthritis are not exposed to PS (PS-PMP) [25,26]. The exact molecular mechanisms of PMPs not exposed to PS are unclear and may involve a specific phospholipase or enzyme that maintains the asymmetry of the PMP membrane [27]. Another explanation is that PS is indeed exposed on all PMP surfaces. However, multiple factors (such as insufficient PS expression and membrane bending) hinder the detection of PS-binding probes (such as AnnexinV and Lactherin) [28]. 

PMPs also express platelet-derived glycoproteins (GP), such as GPIIb/IIIa (CD41/CD61), and activation markers, such as P-selectin (CD62P) [29]. In addition to the heterogeneity of surface markers, PMPs are highly diverse in content [30]. In addition to the active enzymes such as cyclooxygenase-1 (COX-1) and 12-lipoxygenase (ALOX12), they also contain coagulation factors and immune mediators [29]. A protein omics study revealed the correlation between the content and size of PMPs: a small PMP (100–500 nm) is rich in proteins, especially proteins from α particles. Additionally, large PMPs are rich in lipid mediators and mitochondrial proteins [31]. A recent study showed that PMP-containing mitochondria with complete functions could be released from platelets during activation. A purification method was used to produce mitochondria with high purity and integrity from platelets [32]. In addition to organelles, PMPs are rich in platelet-derived cytokines, enzymes, mitochondrial DNA (mtDNA), RNA (e.g., mRNA, miRNA, lncRNA, circRNA), and even transcription factors [33,34] (Figure 1).

## 3. PMPs and Autoimmune Diseases

### 3.1. PMPs and Rheumatoid Arthritis

Rheumatoid arthritis (RA) is a chronic, inflammatory, autoimmune disease that can cause synovitis and destructive arthritis, accompanied by manifestations of extraarticular diseases [35]. RA is not a genetic disease; however, its pathogenesis may be related to genetic factors [36]. Current studies suggest that genetic factors cause susceptibility to rheumatoid arthritis, and environmental factors (such as viruses and drugs) induce the occurrence of RA [36,37]. As the disease develops, rheumatoid arthritis may cause bone destruction and joint deformities [38]. If not controlled, it will accelerate the development of atherosclerosis and shorten the life span, and become a great burden to individuals with the disease [38]. Platelets play an important role in RA, and it has been proven that activated platelets can also participate in the pathological processes of RA by producing PMPs [39,40,41]. Compared to control groups, the PMP content in patients with RA is significantly higher than that in healthy individuals [26,42]. PMPs mainly amplify the inflammation of rheumatoid arthritis by stimulating synovial cells to release activating cytokines IL-6 and IL-8 into the joint space [38,43]. In addition, studies have shown that the antigens citrullinated fibrinogen and vimentin on the PMP surface interact with autoantibodies present in the joints of RA patients, triggering neutrophils to produce pro-inflammatory leukotrienes [44,45]. Therefore, blocking PMPs carrying the antigens citrullinated fibrinogen and vimentin is a potential strategy for the treatment of RA [44,45]. Recent research has shown that RA mainly promotes an inflammatory response in vivo through PMP-induced monocyte activation [46]. Under conditions of blood flow, P-selectin on the surface of PMPs binds to monocytes, which leads to its activation and promotes the occurrence of inflammation [38,47]. PMPs can also activate neutrophils, further supporting the role of PMPs in inflammation [38,44]. Some studies have found that some chemokines (such as C-C motif chemokine ligand (CXCL) 5, CXCL4, and CXCL7) can be transmitted to synovial cells through PMPs, and promote synovitis and joint erosion through the NF-κB pathway [48]. In addition, the formation of PMPs may also be initiated by the complement factors Bb, C4d, and C5B-9, however, the specific mechanism needs to be studied further [44]. In all studies on patients with RA, an increase in PMPs was related to disease activity [49]. In addition to inflammation, PMPs are associated with thrombotic disease in patients with RA [49]. PMPs form dense fibrin clots through close interaction with fibrin fibers, which leads to cardiovascular diseases in patients with RA [50]. Studies have shown that Rac1-specific deleted (Rac1^−/−^) platelets or Rac1-specific inhibitor NSC23766 can significantly inhibit the formation of PMPs. Therefore, Rac1 inhibitors can reduce the release of PMPs, alleviate the inflammatory reaction of RA, and reduce the risk of thrombosis [51].

### 3.2. PMPs and Systemic Lupus Erythematosus

Systemic lupus erythematosus (SLE) is an autoimmune inflammatory connective tissue disease involving multiple organs that occurs mostly in young women [52]. At present, its pathogenesis is not completely clear, mainly due to genetic and environmental factors [53]. It has been reported that SLE is related to HLA-DR2 and DR3 in the North American Caucasian population [54]. In addition, environmental factors (drugs, viruses, etc.) may lead to the occurrence of SLE [55,56]. While continuously releasing autoimmune antibodies, MPs will also be continuously released, so they will aggravate the spread of the disease in SLE patients, and the immune complexes produced will continue to accumulate in the blood vessels throughout the whole body, causing damage to various organs [57,58]. Studies have shown that inflammatory factors play a key role in SLE pathogenesis [59]. High expression of CD41 was found in PMPs released from platelets in SLE patients [59]. After an in-depth study, it was found that PMPs with high expression of CD41 secreted leukotrienes after being internalized by neutrophils [59]. Meanwhile, PMPs promote joint inflammation by releasing IL-1β to increase the levels of IL-6 and IL-8 in fibroblasts [59]. Studies also have found that IgG on the PMP surfaces are associated with SLE activity, as monocytes from SLE patients bind to and internalize IgG+ PMP. The expression of CD69, CD64, IL-1β, TNF-α, and INF-α can be promoted by changing the phenotype of monocytes [60]. In summary, in SLE patients, due to the damage to platelets and other cells caused by autoantibodies, the PMPs released by the damaged platelets will secrete inflammatory factors by stimulating inflammatory cells such as neutrophils and mononuclear cells, eventually leading to tissue or organ damage [59,60]. Patients with SLE are also relatively more prone to cardiovascular diseases. Through a retrospective experiment, it was found that SLE patients showed a high level of PMPs, patients with high PS+PMPs had a higher incidence of thrombotic events in the past, and the expression of P-selectin was related to disease activity [61]. In addition, multiple organ damage caused by the excessive deposition of pathological immune complexes is a clinical manifestation of SLE. Different autoantigens (such as the non-histone nuclear protein high mobility group (HMGB1) and nucleic acid) carried by PMPs contribute to the formation of an immune complex [60]. According to related research reports, the anti-dsDNA antibody commonly found in SLE patients recognizes platelet glycoprotein IIIa (CD61), which explains why SLE patients have high concentrations of PMPs [60]. In addition, miRNA and mtDNA in PMPs may also be a source of nucleic acid autoantigens [60]. Therefore, if the autoantigens are known, blocking the autoantigens carried by PMPs is a potential strategy for treating SLE [60].

### 3.3. PMPs and Antiphospholipid Antibody Syndrome

Antiphospholipid syndrome (APS) is a disorder characterized by recurrent arterial or venous thrombosis, morbid pregnancy, and persistent positive antiphospholipid antibodies (APL) [62,63]. APS can be secondary to systemic lupus erythematosus or other autoimmune diseases, but can also occur alone (primary APS), in which case it is a systemic autoimmune disease [64]. The family tendency of APS is not obvious, but the antiphospholipid antibody test on relatives of patients can often be positive [62,63]. Based on genetic factors, certain infections and drug inducements can lead to APS [65]. Clinically, APS patients mainly present with thrombocytopenia, autoimmune hemolytic anemia, vascular embolism, and abortion In severe cases, extensive thrombosis may lead to death [66]. At present, many studies have reported on the mechanisms of PMS leading to APS occurrence. According to previous studies, the elevated expression of P-selectin on the surface of the PMPs in individuals with APS indicates the activation of platelets, thereby suggesting a heightened risk of acute thrombotic events [67,68]. PMPs in patients with APS have been found to increase the expression of TNF-α, ICAM-1, and VCAM-1, inhibit angiogenesis, and promote endothelial cell apoptosis by recruiting monocytes [69]. Recent studies have shown that anti-β2 glycoprotein (GPI)/β2GPI complex antibody is related to the abnormal activation of PMPs, which can lead to inflammatory necrosis of endothelial cells and will eventually lead to autoimmune system damage [70]. Di et al. proved that PMPs induced by anti-β2GPI/β2GPI complex leads to cell necrosis through NLRP3/NF-κB/Gasdermin D (GSDMD) and NLRP3/Caspase-1 signaling pathways [70]. Inhibition of NLRP3 expression in PMPs effectively reduces inflammatory reactions and endothelial cell death. These studies provide important insights into how PMPs lead to compromised immune systems in patients with antiphospholipid antibody syndrome and suggest potential options for future treatment [70]. However, to predict the risk of thrombosis in patients with APS in advance, Jiang et al. applied a nano-heterojunction photoelectrochemical biosensor to realize ultra-sensitive detection of molecular markers in the PMPs of APS patients [71]. Team Jiang found that there was a high level of LncNR_040117 expression in the PMPs, which led to biological phenomena such as apoptosis by activating the MAPK signaling pathway [71]. Jiang et al. developed a photoelectrochemical biosensor based on a β-In2S3@g-C3N4 nano-heterojunction, which realized the ultrasensitive detection of LncNR_040117 [71]. LncNR_040117 in PMPs has been identified as an effective biomarker of APS, and is expected to provide a reliable diagnostic platform for RM/APS using biosensor technology. For the LncNR_040117 carried by PMPs, researching the corresponding drugs for targeted blocking could become a potential method to block the disease process.

### 3.4. PMP and Sjogren Syndrome

Sjogren syndrome (SS) is a chronic inflammatory autoimmune disease, also known as autoimmune exocrine gland epithelial inflammation or autoimmune exocrine disease [72]. SS is mainly caused by a combination of heredity and the environment [73]. Based on genetic factors, if the patient’s resistance decreases or is infected with certain viruses, such as EB virus, Coxsackie virus, retrovirus, hepatitis C virus, and HIV virus, SS can occur [74]. Clinically, in addition to salivary glands and lacrimal glands being damaged, resulting in dry mouth and eyes, other exocrine glands and other organs outside the glands are involved, resulting in multi-system damage symptoms [72]. Patients with SS may develop thrombocytopenia, accompanied by anemia and leukopenia [72]. This is because SS patients can form various autoantibodies, some of which target the blood system [75]. The destruction of a large number of platelets may lead to an increase in PMPs. Some studies detected the levels of plasma in total MPs, PMPs, and white blood cell MPs in patients with SS using flow cytometry and found that the levels of PMPs in patients with SS increased [76,77]. In patients with severe SS, leukocyte MPs also increase [76,77]. The increase in soluble CD40 ligand (sCD40L) and soluble P-selectin is a manifestation of platelet activation, and PMPs in patients with SS also showed high expression of sCD40L and soluble P-selectin [76,77]. Elevated PMP levels in patients with SS reflect the activation status of systemic cells, especially platelets, which also explains why patients with SS are more prone to thrombosis [76,77]. After collecting a significant amount of clinical data, it was found that the levels of PMPs in SS patients were negatively correlated with the levels of serum sPLA2 and β2 microglobulin [76,77]. Clinically, PMP content can be determined indirectly by monitoring serum sPLA2 and β2 microglobulin levels. Therefore, many scholars suggest combining PMPs with other clinical indicators to reflect disease progression and treatment of patients with SS [76,77]. However, the specific mechanism by which PMPs lead to disease progression in patients with SS remains unclear.

### 3.5. PMPs and Systemic Sclerosis

Systemic sclerosis (SSc) is an autoimmune disease of unknown etiology, and its pathogenesis is related to genetic and environmental factors [78]. Certain drugs such as bleomycin can induce epidermal fibrosis in patients [79]. In addition, viral infections, such as cytomegalovirus, and Epstein-Barr virus, can cause the onset of SSc [80,81]. As the disease progresses, patients are predominantly characterized by peripheral microvascular injuries, immune system activation, and extensive skin fibrosis, which are common in women [82]. Endothelial cell injury leads to the continuous activation of platelets and fibrosis of the skin, and MPs can be used as biomarkers of endothelial injury [83,84,85]. Oyabu et al. used ELISA to compare the PMP levels in patients with four different autoimmune diseases, including SSc patients [86]. The results showed that the level of PMPs in SSc patients was significantly higher than that in the control group [86]. The clinical data in different regions also confirmed this result, that is, there is a high concentration of PMPs in patients [86,87,88]. Therefore, different researchers suggested that PMPs should be used as molecular detection markers for SSc [86,87,88]. Under normal physiological conditions, the phagocytosis of platelets directly restricts the release of PMPs into the blood, thus preventing unnecessary systemic diffusion. This steady-state mechanism depends on the interaction between P-selectin and its receptor PSGL-1; however, the receptor is damaged in patients with SSc. In addition, it was also found that microvascular damage in SSc patients is related to autophagy [89]. Recently, different research groups found that PMPs in the blood of patients with SSc highly expressed mobility histone B1 (HMGB1) related to the damage-related molecular pattern (DAMO) [89]. HMGB1+PMP interacts with neutrophils and promotes autophagy, which is characterized by an increase in neutrophil extracellular traps (NET) [89]. This indicates that autophagy of neutrophils is related to endothelial injuries and the fibrosis of SSc, and HMGB1+PMP can be a potential detection index and candidate marker for therapeutic targets of SSc [89]. Recently, it was proposed that PMP-containing mtDNA (such as MT-ATP6) can promote the occurrence of interstitial lung disease in SSc patients by activating the inflammatory immune response of fibroblasts [87,88]. Studies have found that methotrexate can affect PMP content and especially the DNA load [87]. Therefore, the author proposed a potential treatment plan for patients with SSc [87]. Alternatively, it may be more effective to block the occurrence of interstitial lung disease in SSc patients specifically by sexually inhibiting the content of PMPs carrying a specific mtDNA (such as MT-ATP6) locally.

### 3.6. PMPs and Ankylosing Spondylitis

Ankylosing spondylitis (AS) is an autoimmune disease that belongs to the rheumatism category [90,91]. The etiology of AS is unclear, and genetic factors play an important role in its pathogenesis. It is generally believed that it is directly related to HLA-B27 [90]. The incidence of AS in HLA-B27 positive patients ranges from 10% to 20% [90]. Immune factors are also a cause [91]. The study found that the levels of complement C4 and IgA antibodies in the serum of patients with AS were significantly increased [91]. Trauma, endocrine disorders, metabolic disorders, and allergies can also cause the disease [92]. AS generally occurs in young men [93]. As the disease progresses, it mainly manifests as spinal rigidity and fibrosis, and can cause different degrees of lesions in the eyes, lungs, muscles, and bones [93]. Platelets play an essential role in the occurrence and development of AS [94]. However, the relationship between platelet derivatives and AS remains unclear. An analysis of relevant research shows that PMPs do not correlate with disease activity, function, or spinal mobility indices in AS patient who did not have classical cardiovascular risk factors [94]. However, significantly downregulated PMPs in patients receiving anti-tumor necrosis factor (anti-TNF) therapy suggests that anti-TNF therapy may have a beneficial effect on vascular function in AS [95]. Recently, Hong et al. conducted a transcriptome sequencing and bioinformatic analysis of AS and healthy control platelets [96]. According to GO analysis results, the biological processes of differentially expressed mRNAs mainly include platelet degranulation and vesicle-mediated transport [96]. According to KEGG analysis, differentially expressed mRNAs are mainly involved in the regulation of platelet activity, gap junction, focal adhesion, and regulation of the actin cytoskeleton [96]. Therefore, according to the GO and KEGG pathway analysis results, platelets in AS patients are involved in platelet activation, degranulation, and platelet-related vesicle transport, suggesting that PMPs may be involved in the immunomodulation of AS [96]. In addition, the author also found some signaling pathways involved in the occurrence and development of AS, including Rap1, Hippo, AMPK, MAPK, and PI3K-Akt, all of which are involved in immune regulation and the inflammatory response [96]. Therefore, AS patients may transmit inflammatory and immune signals to recipient cells through PMPs and ultimately promote the development of the disease; however, the specific mechanism remains to be studied.

### 3.7. PMPs and Systemic Vasculitis

Systemic vasculitis (SV) is a group of immune diseases characterized by inflammation and destruction of blood vessels, involving large, medium, and small vessels throughout the body [97]. The etiology of SV is still unknown and is mainly influenced by heredity and the environment [97]. SV can affect any organ system in the body, including the kidneys, lungs, peripheral and central nervous system, heart, eyes, musculoskeletal systems, and skin [98]. If not detected and treated early, SV can lead to associated complications and even death [99]. SV is more common in adolescents [100]. Brogan et al. reported that children with active SV had significantly higher levels of PMPs and endothelial MPs than controls [101]. It was concluded that PMP levels were strongly correlated with disease activity [101,102]. The most recent study reached the same conclusion and demonstrated that PMP levels were significantly correlated with inflammation and renal injury [103]. Through an in-depth mechanistic study, it was found that the increase in chemokines, adhesion factors, growth factors, and apoptosis factors in PMPs ultimately play an important role in endothelial destruction and vasculitis via the NF-κB pathway [103]. In vasculitis, PMPs are mainly used as a medium to spread proinflammatory factors in an all-encompassing manner. Therefore, the author suggests that preventing the spread of PMPs can be used as a potential clinical treatment scheme [103]. Kawasaki disease (KD) is an acute systemic vasculitis in children [104]. KD treatment should effectively control vascular inflammation and prevent coronary artery dilatation. The standard treatment is intravenous immunoglobulin (IVIG) and oral aspirin, which can significantly reduce the incidence of coronary artery injuries [104]. Jin et al. found that the PMP levels of children with vasculitis was significantly higher than that of the control group before IVIG [105]. After IVIG, the level decreased significantly and reached the lowest level after 1–2 months of treatment, but some patients rebounded and developed drug resistance. The authors found that the average PMP level in patients with IVIG resistance was significantly higher than that in the control group, and therefore the authors concluded that a high PMP level may be one of the mechanisms of IVIG resistance. In addition, PMPs are positively correlated with inflammatory factors such as C-reactive protein, IL-6, and sIL-2R levels [105]. An in-depth study found that PMPs can directly induce monocytes, macrophages, neutrophils, and vascular endothelial cells to express tissue factors and promote thrombosis [105]. Therefore, many scholars suggest that PMPs can be used as a biomarker to monitor the progression of vascular inflammation and the risk of thrombosis in patients with SV [105].

## 4. Summary

Increasing evidence supports PMPs as a medium that participates in intercellular communication, inducing inflammation, immune stimulation, immunosuppression, and even thrombosis. The contents and surface markers of PMPs have varying manifestations in different types of immune diseases, but the overall change trend of PMPs is increased (Table 2). Therefore, PMPs have great potential for detecting and monitoring the occurrence and development of autoimmune diseases. In this review, we provide evidence that PMPs can be used as a biomarker of several autoimmune diseases, and briefly summarize the potential pathogenesis of PMPs in different autoimmune diseases according to the current research status, which provides a basis for the diagnosis, monitoring, and treatment of diseases. However, basic research on PMPs is still in its early stages, and the lack of in-depth research on their mechanisms hinders their clinical application. Therefore, before PMPs are applied to the clinical diagnosis, monitoring, and treatment of autoimmune diseases, it is necessary to conduct more in-depth research on PMPs, including (i) the isolation and purification of PMPs; (ii) in-depth understanding of the occurrence and targeting of PMPs; (iii) in-depth study of the mechanisms of inflammatory reactions, immune reactions, and thrombosis caused by PMPs; (iv) research to evaluate the effectiveness and reliability of PMPs as nano-drugs or in vivo drug delivery systems; and (v) clinical application research. Although PMPs have challenges and difficulties to overcome before clinical application, their biological and physiological characteristics show great potential as a biomarker and therapeutic tool. In a word, an in-depth study on the physiological function and related mechanism of PMPs is helpful to promote the clinical transformation of PMPs.

## Figures and Tables

**Table 1:** Differences between platelet derived exosomes and PMPs.

	Exosomes	PMPs
Size	30~150 nm	100~1000 nm
Density	1.14–1.18 g/mL	>1.23 g/mL
Shape	Relatively uniform, round cup shape	Different sizes and shapes
Origin	Multivesicular bodies (MVB)	Plasma membrane
Release mechanism	Exocytosis	Ectocytosis
Markers	Tetraspanins Heat shock proteins ALG-2-interacting protein X Tumor susceptibility protein Class I and class II major histocompatibility complex	Phosphatidylserine (PS) P-Selectin CD31 GPIIb/IIIa (CD41–CD61) Other antigens of parental cell
Reference	[15,16,17]	[20,21,22]

**Table 2:** The association of PMPs with autoimmune disorders.

Autoimmune Disease	In-/Decrease of PMP	PMP Associated Molecules	Pathogenic Role of PMP	References
Rheumatoid arthritis	increase	P-selectin (CD62P), citrullinated fibrinogen, vimentin, CXCL5, CXCL4, and CXCL7	PMP induces its activation and secretes inflammatory factors by binding with monocytes and neutrophils, and finally promotes the occurrence of inflammation. The fibrin clot formed by PMP through its interaction with fibrin fibers leads to vascular embolism. Chemokines are delivered to recipient cells through PMP, and inflammation is promoted through NF-κB pathway.	[38,39,40,41,42,43,44,45,46,47,48,49,50,51]
Systemic Lupus Erythematosus	increase	CD41, CD61, P-selectin, IL-1β, IgG, and HMGB1	PMP with high expression of CD41 and CD61 can induce neutrophils to release leukotrienes, fibroblasts to release IL-6 and IL-8, and finally promote inflammation. Autoantigens (HMGB1, CD61, etc.) on the surface of PMP react with specific antibodies to produce immune complexes which are deposited in organs, resulting in organ damage.	[59,60,61]
Antiphospholipid antibody syndrome	not statistically significant	TNF-α, ICAM-1, VCAM-1, anti-β2GPI/β2GPI complex, and LncNR_040117	Resistance of PMP surface β 2GPI/ β2GPI complex passes NLRP3/NF-κB/GSDMD signal pathway and NLRP3/Caspase-1 signal pathway lead to inflammatory necrosis of endothelial cells. LncRNA_040117 is transmitted to endothelial cells through PMP, activating MAPK signaling pathway leads to cell apoptosis.	[67,68,69,70,71]
Sjogren syndrome	increase	To be studied	To be studied	[75,76,77]
Systemic sclerosis	increase	P-selectin, HMGB1, and mtDNA (MT-ATP6)	Accumulated HMGB1+ PMP in vivo interacts with neutrophils to promote their autophagy and eventually lead to vascular endothelial injury and fibrosis. MtDNA (such as MT-ATP6) contained in PMP is internalized by fibroblasts, which promotes its inflammatory immune response and leads to interstitial lung disease.	[86,87,88,89]
Ankylosing Spondylitis	not statistically significant	To be studied	AS patients may transmit inflammatory and immune signals through PMP, and ultimately promote the development of the disease.	[95,96]
Systemic vasculitis	increase	chemokines, adhesion factors, growth factors, and apoptosis factors	The proinflammatory cytokines carried by PMP lead to inflammatory reaction of cells through NF-κB pathway, which eventually leads to endothelial destruction and vasculitis. PMP can directly induce monocytes, macrophages, neutrophils and vascular endothelial cells to express tissue factors and promote thrombosis.	[98,99,100,101,102,103,104,105]

## Data Availability

Data sharing not applicable. No new data were created or analyzed in this study. Data sharing is not applicable to this article.
